# Supplementary material for: Ba+2 ion trapping using organic submonolayer for ultra-low background neutrinoless double beta detector
Source: Nat Commun. 2022 Dec 14;13:7741. doi: 10.1038/s41467-022-35153-0 (PMC9750972; doi:10.1038/s41467-022-35153-0)
Supplement: Supplementary file 1 — Supplementary Information [file 41467_2022_35153_MOESM1_ESM.pdf]

# Supplementary Information

# $Ba^{2+}$ ion trapping using organic submonolayer for ultra-low background neutrinoless double beta detector

P. Herrero-Gómez[1,2], J.P. Calupitan[1], M. Ilyn[1], A. Berdonces-Layunta[1,2], T. Wang[1,2], D. G. de Oteyza[1,2], M. Corso[1,2], R. González-Moreno[1,2], I. Rivilla[2,3], B. Aparicio[4], A.I. Aranburu[5], Z. Freixa[3,5], F. Monrabal[2,3], F.P. Cossío[2,4], J.J. Gómez-Cadenas[2,3], C. Rogero[1,2*] and NEXT collaboration.

[1]Centro de Física de Materiales (CSIC-UPV/EHU), San Sebastián, E-20018, Spain.
[2]Donostia International Physics Center (DIPC), San Sebastián, E-20018, Spain.
[3]Ikerbasque, Basque Foundation for Science, Bilbao, E-48009, Spain.
[4]Department of Organic Chemistry I, Centro de Innovación en Química Avanzada (ORFEO-CINQA), University of the Basque Country (UPV/EHU), San Sebastián, E-20018, Spain.
[5]Department of Applied Chemistry, University of the Basque Country (UPV/EHU), San Sebastián, E-20018, Spain.

*Corresponding author(s). E-mail(s): celia.rogero@csic.es;

## Supplementary Note 1 - Molecules inside the molecular islands

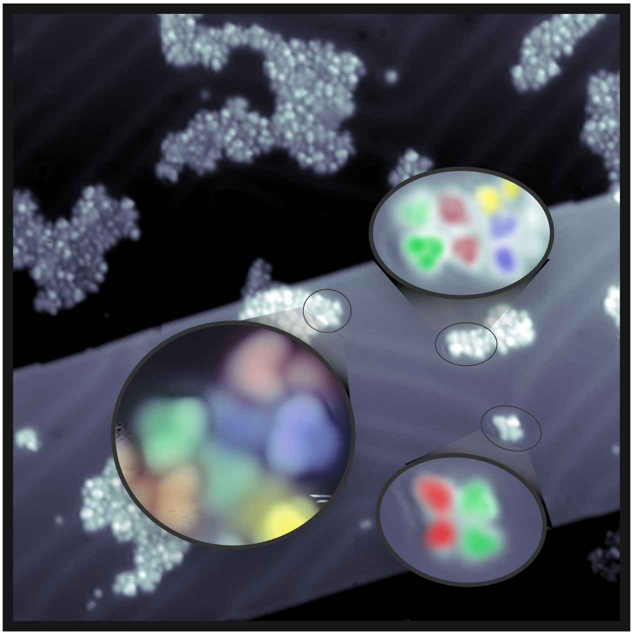

**Supplementary Fig. 1**: Several molecular aggregates have been highlighted in the large scale image shown in Figure 3 of the main text. As a guess of the molecules inside the aggregates, we use fictitious colors (each molecule corresponds to a colour). This implies that no molecular fragments are evaporated in addition to the intact molecules. This is also supported by the fact that the XPS stoichiometries remained constant over the course of the several (over 10) evaporations performed for this study.

# Supplementary Note 2 - Simulation of the most stable molecular conformation upon chelation with $BaCl_2$

In our previous manuscript [1] we presented the calculations for the chelation of the FBI molecule with Ba ions surrounded by Xe atoms as well as by Ba perchlorate. Both calculations demonstrated that the most stable configuration was that where the Ba ions ended in the presenting coordination bonds with N, phenyl ring and O atoms. Based on them, we compute the chelation of the FBI with $BaCl_2$. As starting point we located the $BaCl_2$ in the surroundings of the crown ether and we leaved the system to evolve to the most stable configuration. As it can be seen in Figure 2a, the final geometry resembles that calculated before. The N···$Ba^{2+}$, Ph···$Ba^{2+}$ and crown ether···$Ba^{2+}$ bonds are preserved on passing from naked $Ba^{2+}$ cation to $BaCl_2$ salt. These geometries show a computed root-mean-square deviation value of RMSD = 2.83 Å, excluding the two chloride anions.

We have also computed another coordination pattern, forcing the Barium cation to interact with the sensor from the outside, namely through the convex face of the crown ether connected to the fluorophore. In this case, the ended interacting only with the crown ether forming a convex complex, as it is shown in Figure 2b. In this case, all the O···$Ba^{2+}$ distances are very similar and lie in the 2.83 - 2.87 Å range. The only N···$Ba^{2+}$ interaction exhibits a slightly larger distance of 3.13 Å. Therefore, this complex has a more restricted coordination pattern since both Ph···$Ba^{2+}$ and N···$Ba^{2+}$ are not available. Consequently, the convex complex is 7.4 kcal/mol (6.9 kcal/mol if Gibbs energies at 298 K are considered) less stable than the previously mentioned concave complex. This means that, thermodynamically, the latter should be ca. $3.0 \times 10^5$ more abundant than the latter. However, if the sensor is adsorbed on the surface and the concave face is kinetically less accessible, perhaps the formation of a

minor amount of the convex complex cannot be completely ruled out, although even in this rare event the metastable convex complex should evolve towards the thermodynamically more stable concave one.

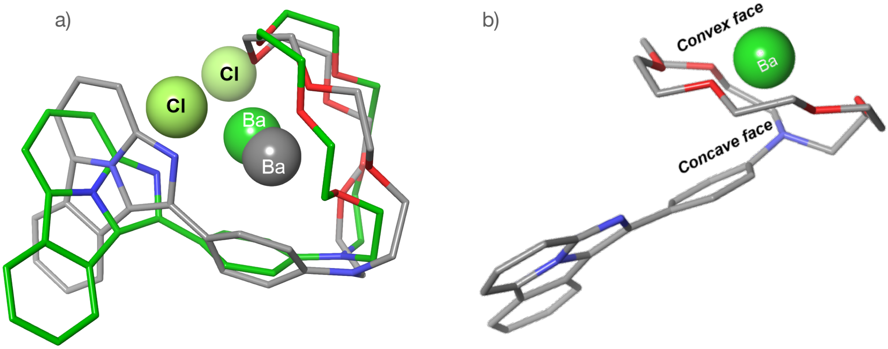


**Supplementary Fig. 2**: Graphical representations ofthe RMSD associated with fully optimized geometries (B3LYP-D3BJ/6-31G*&LANL2DZ level of theory) of: a) FBI molecule coordinated to a naked Ba2+ cation (gray) and to BaCl2 (green); b) FBI molecule coordinated to a naked Ba2+ along the convex face of the crown ether. Hydrogen atoms are not shown.

# Supplementary Note 3 - Evolution of O 1$s$ and Ba $3d_{5/2}$ core levels with increasing $BaCl_2$ dosage

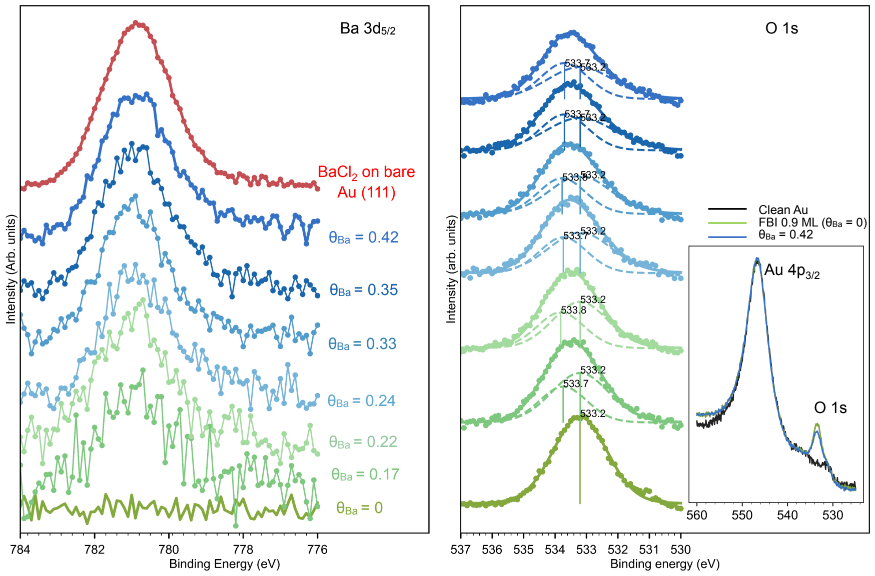


**Supplementary Fig. 3**: O 1$s$ (right) and Ba $3d_{5/2}$ (left) core levels evolution measured for FBI on Au(111) with subsequent evaporation of $BaCl_2$ (data on Fig. 2e). The Ba $3d_{5/2}$ core level is always centered on 780.9 eV. The spectrum of $BaCl_2$ directly deposited on bare Au(111) also appears centered on 780.8 eV where the Ba is in the +2 oxidation state. Inserted is the Spectral region around the Au $4p_{3/2}$ and O 1$s$ core levels. Spectra corresponds to clean Au (111), 0.9ML of FBI, FBI after chelation with 0.42 $Ba^{2+}$ ions per molecule.

# Supplementary Note 4 - Conformational simulations of free and chelated FBI with $Ba^{++}$ and $Na^{+}$

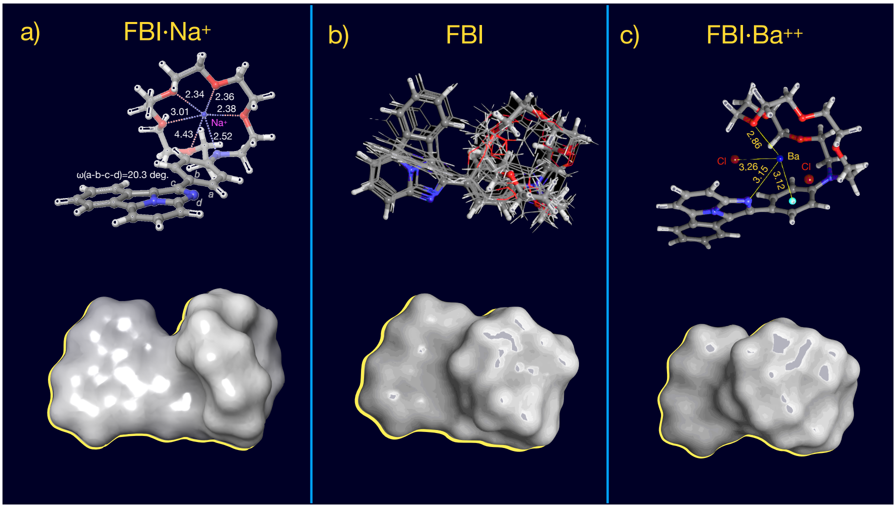


**Supplementary Fig. 4**: Fully optimized structure (top) and the electron density surface associated with the most stable conformer (bottom) for: a) FBI·$Na^{+}$ complex computed at the B3LYP-D3BJ/6-311++G(d,p)&LANL2DZ level of theory. The cation $Na^{+}$ interacts only with the crown-ether, which bends slightly. Since this cation is smaller than $Ba^{2+}$, it does not occupy all the coordination positions, i.e. it does not interact with the phenyl ring nor with the nitrogen from the fluorophore; b) MM–MC conformational analysis (OPLS3e force field) of FBI. The Ball & Stick representation corresponds to the most stable conformation. The other structures correspond to the ten most stable conformers within 0.0-4.8 kJ/mol; c) fully optimized structure of FBI·$BaCl_2$ complex computed at the B3LYP-D3BJ/6-311++G(d,p)&LANL2DZ level of theory. Bond distances are given in Å.

# Supplementary Note 5 - STS measurements for un-chelated and chelated molecules

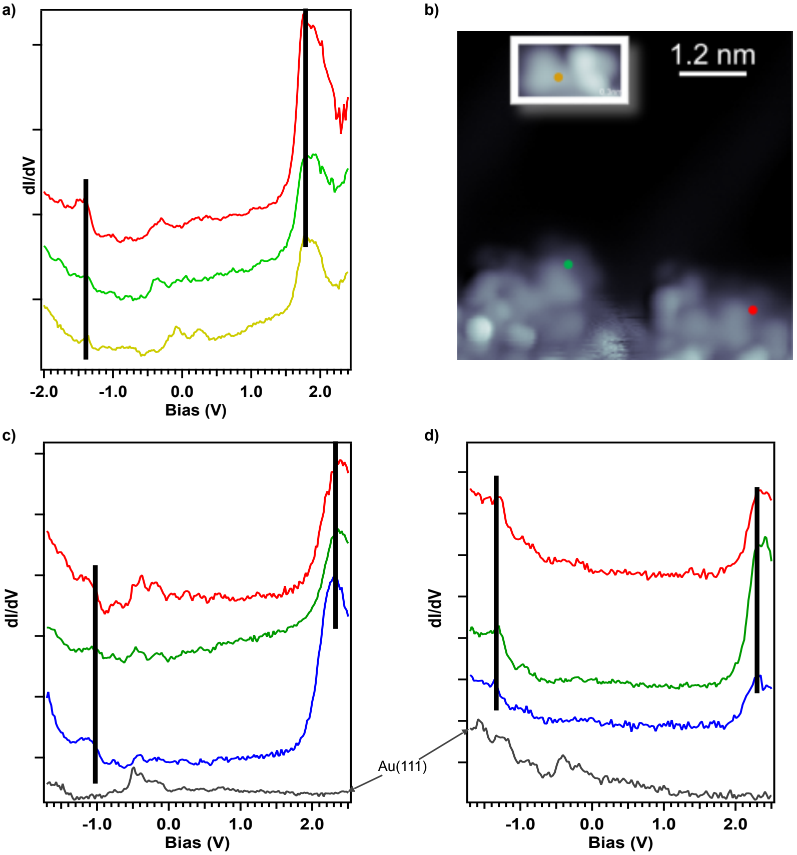


**Supplementary Fig. 5**: a) Comparison of STS measured on the molecular islands (red, green) and on an isolated molecule (orange). b) STM image of the area where the spectra of molecular islands where measured (the isolated molecule was included as an inset). The green, red and orange spots at the STM image indicate the points where the STS spectra were recorded. c) and d) STS measured on different isolated molecules upon chelation with $Na^{+}$ and $Ba^{2+}$.

## Supplementary Note 6 - O *1s* evolution for low and near-saturation dose of Ba$^{2+}$ on Cu(111)

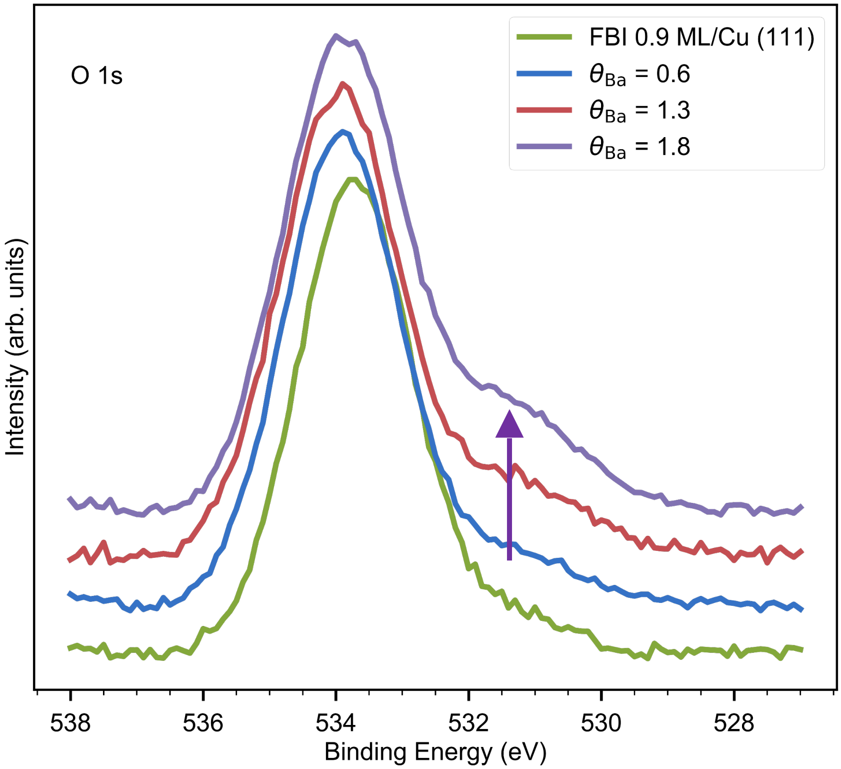


**Supplementary Fig. 6**: O 1*s* core level of FBI on Cu (111) (green) and FBI-$BaCl_2$ in different doses. The shift toward higher BE is still present for these concentrations of Ba$^{2+}$ (blue arrow). The shoulder at low BE (531 eV) from $Cu_2O$ contamination grows with the exposure to the evaporator (purple arrow). This is due to the higher concentration of oxygen in the evaporation module, which had a slightly higher pressure than the characterization module.

## Suplementary Reference

Rivilla, I. *et al.* Fluorescent bicolour sensor for low-background neutrinoless double $\beta$ decay experiments. *Nature* **583** (7814), 48–54 (2020).
